# Hospital surveillance of influenza strains: a concordant image of viruses identified by the Swiss Sentinel system?

**DOI:** 10.1111/irv.12417

**Published:** 2016-08-31

**Authors:** Ana Rita Gonçalves, Anne Iten, Patricia Suter‐Boquete, Manuel Schibler, Laurent Kaiser, Samuel Cordey

**Affiliations:** ^1^ Swiss National Reference Centre for InfluenzaGeneva University HospitalsGenevaSwitzerland; ^2^ Laboratory of VirologyGeneva University HospitalsGenevaSwitzerland; ^3^ University of Geneva Medical SchoolGenevaSwitzerland; ^4^ Infection Control Program and WHO Collaborating Centre on Patient SafetyGeneva University HospitalsGenevaSwitzerland

**Keywords:** community, hospital, influenza, surveillance, Switzerland

## Abstract

**Background:**

The Swiss Sentinel system for influenza virus surveillance reports influenza‐like illness in the community through a network of primary care practitioners, but the epidemiologic, demographic, and virological characterization may differ from that observed in hospitalized patients with influenza.

**Objective:**

To compare demographic and virological data from hospital influenza cases with Sentinel system data during the 2014–2015 season.

**Methods:**

We included 2623 in‐ and outpatients with a screening request for influenza A/B in a university teaching hospital in Geneva, Switzerland, and 933 participants from the Swiss Sentinel surveillance system and compared the demographic and virological data of the two populations, including the respective distribution of influenza subtypes, and conducted a phylogenetic comparison at the HA1 level of influenza viruses recovered in community and hospital cases.

**Results:**

There were similar proportions of influenza strains recovered in the hospital and in the community (H3N2, 57.1% and 56.9%; H1N1pdm09, 15.5% and 14.2%; B, 27.4% and 28.8%, respectively). HA1 sequence analysis confirmed that all three strains were genetically similar between the two populations. During this particular season, influenza cases were detected earlier in the hospital than in the Sentinel system.

**Conclusions:**

Although an influenza surveillance system based on the community can predict the type of influenza strains that will be associated with hospitalizations, it fails to estimate the potential virulence of circulating strains. Further, the population characteristics in the community differ from those in hospitalized patients. This suggests that any national influenza surveillance system should include both community‐ and hospital‐based surveys.

## 1 Introduction

Influenza virus infections are a major burden worldwide in terms of human morbidity, mortality, and public health costs.^1^, ^2^ In Switzerland, a network of primary care medical practitioners (Sentinel surveillance system) reports medical consultations for influenza‐like illness (ILI) on a weekly basis to the Federal Office of Public Health (FOPH). A subgroup of these practitioners randomly collects respiratory samples from patients diagnosed with ILI for influenza virus detection and characterization at the Swiss National Reference Centre for Influenza (NRCI) in Geneva. In addition, it is compulsory to report influenza A and B infections diagnosed by hospitals and private laboratories to the FOPH.^3^, ^4^, ^5^

The ILI‐based Swiss Sentinel surveillance of Influenza monitors the evolution of influenza activity and the duration of the influenza season. However, it cannot identify severe acute respiratory infections due to influenza requiring hospitalization, often observed in individuals with underlying chronic conditions and the elderly. A hospital‐based sentinel surveillance would fill this gap, complementing the actual influenza compulsory reporting by hospitals, by providing important data on high‐risk groups for influenza infection, for which prevention and treatment should be prioritized. This would permit a better understanding of the clinical features of influenza infection in hospitalized patients with a challenged health status including associated comorbidities and mortality rates and provide a more accurate estimation of the global burden of the disease.^6^, ^7^ Characterizing hospital‐based influenza strains would also provide the opportunity to assess whether these strains mirror those circulating in the community, increasing the probability to identify more virulent isolates. This strategy is supported by the increasing adherence to international or country‐specific, hospital‐based surveillance systems by several countries.^6^, ^7^, ^8^, ^9^

The aim of this study was to compare demographic, epidemiological, and in particular, virological data from hospital‐based influenza cases with data collected by the Swiss Sentinel system during the 2014–2015 influenza season.

## 2 Methods

### 2.1 Setting and individuals/samples included

We conducted a retrospective study at the Geneva University Hospitals, Geneva, Switzerland, where inpatients and outpatients are routinely screened for influenza A/B for medical follow‐up or diagnostic purposes. Demographic, epidemiological, and virological data from hospital patients were compared to those obtained from the Swiss Sentinel surveillance of influenza. The latter surveillance system relies on the participation of general practitioners, internists, and pediatricians in private practices (n=84 for the 2014–2015 season). Practitioners are requested to collect naso/oropharyngeal swabs from one of five consulting patients with ILI symptoms from week 40 to week 16 of the following year. The practitioners are recruited according to the Swiss population distribution.

*Hospital cases*: All in‐ and outpatients of all ages with at least one respiratory sample (naso/oropharyngeal swab, nasal aspirate, or bronchoalveolar lavage) screened for the presence of respiratory pathogens at the laboratory of virology during the 2014–2015 influenza season (September 29, 2014–April 17, 2015) were included in the study. Among the influenza A‐ and B‐positive clinical isolates, one of five was randomly selected for subtyping and hemagglutinin 1 (HA1) sequencing.

All influenza cases identified among inpatients were prospectively classified as community‐ or hospital‐acquired (nosocomial). A nosocomial case was considered when the onset of ILI symptoms, confirmed by a positive PCR result, occurred more than 48 hours after hospital admission. Nosocomial cases were analyzed separately and not compared to the Sentinel population.

*Sentinel cases:* Individuals consulting Sentinel practitioners and with respiratory samples (naso/oropharyngeal swabs) screened at the NRCI during the 2014–2015 influenza season were included in the study. All influenza‐positive samples were subtyped, and one of five positive samples were submitted for HA1 sequencing.

### 2.2 Virological, genetic, and HA1 phylogenetic analysis of influenza‐positive samples

Viral genomes of samples selected for subtyping and sequencing were individually processed according to the NRCI procedure. Two hundred microliters of the initial respiratory specimens was extracted using the NucliSens easyMAG magnetic bead system (BioMérieux, Geneva, Switzerland) according to the manufacturer's instructions, and viral RNA was recovered in an elution volume of 25 μL. Influenza A‐positive samples were subtyped into H3N2 and H1N1 by real‐time reverse transcriptase polymerase chain reaction (rRT‐PCR) using the One‐Step Supermix (Invitrogen, Carlsbad, CA, USA) in a StepOnePlus^™^ instrument (Applied Biosystems, Rotkreuz, Switzerland). Viral specimens with a cycle threshold value <30 were submitted to HA1 gene sequencing. Prior to sequencing, the extracted viral RNA genomes were used for the synthesis of cDNA using the SuperScript^®^ II Reverse Transcriptase (Invitrogen) with influenza A/B‐specific primers (Table S1). Strain‐specific HA1 cDNAs were further amplified using either a nested PCR for influenza B/HA1 or a first‐round PCR with strain‐specific primers, followed by two independent hemi‐nested PCRs for influenza A/H1N1pdm09 and A/H3N2 HA1, respectively (Table S1). Amplicons were sequenced with strain‐specific primers using conventional Sanger sequencing performed with the ABI 3500xL Genetic Analyzer (Applied Biosystems).^10^ Primer sequences and PCR conditions were applied according to the standard operating procedures of the WHO Collaborating Centre at the National Institute for Medical Research (London, UK). HA1 sequences were analyzed with the software platform Geneious 6.1.6^11^ and aligned using the mafft v7.017 program^12^. Maximum‐likelihood trees were estimated using the PhyML program (1000 bootstrap replicates).^13^ Reference sequences used in the phylogenic trees were imported from the GISAID platform (Table S2).

### 2.3 Data analysis

Differences between groups were tested using the Mann‐Whitney *U* test for continuous variables and the chi‐square test with Yates correction or Fisher exact test for categorical variables using graphpad prism version 6.00 for Windows (GraphPad Software, La Jolla CA, USA). A two‐sided *P* value of <.05 was considered significant. This study was approved by the Research Ethics Committee of Geneva (project # 15‐252‐2015‐00019).

## 3 Results

### 3.1 Demographic, virological, and epidemiologic data

**Figure 1:**
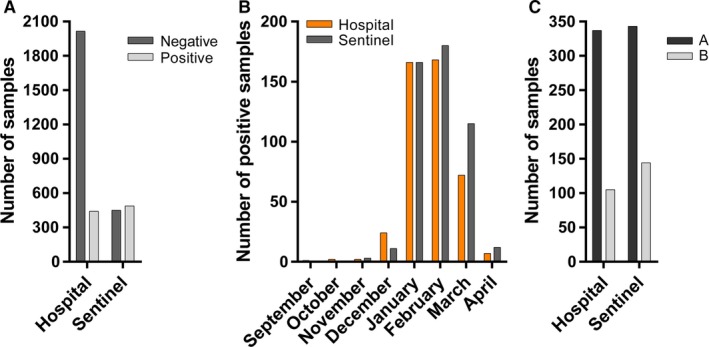
Description of hospital and Sentinel samples. (A) Number of influenza‐positive and influenza‐negative samples for each population. (B) Distribution of influenza‐positive samples throughout the 2014–2015 influenza season. (C) Number of influenza A‐ and B‐positive samples for each population. Nosocomial influenza cases were not included in graphics (A) to (C)

Of the 2623 patients screened for influenza A/B in the hospital, 608 had one positive sample for influenza A or B; one individual had two distinct influenza‐positive samples. Of the 609 (23.2%) influenza‐positive samples, 442 (72.6%) were community‐acquired and 167 (27.4%) were acquired during hospitalization. Of note, nosocomial cases were not taken into account when comparing hospital and Sentinel cases. Of 933 Sentinel screened samples, 487 (52%) were positive for influenza A or B. Four co‐infections were observed in this population, but we report here only the dominant strain. The ratio of positive to negative samples was higher in the Sentinel (1.08) than in the hospital (0.22) population (Table 1; Fig. 1A). The epidemic period, defined as the period during which the number of weekly ILI cases reported to the FOPH was of ≥70 cases per 100 000 inhabitants for 2014–2015 season^4^, lasted from weeks 2–13 (Fig. 1B). The maximal weekly number of positive samples for both populations was reached during the influenza epidemic period (hospital, week 3, 2015; Sentinel, week 6, 2015). Influenza A and B co‐circulated during the 2014–2015 season. Nevertheless, influenza A (76.2% and 70.4%, respectively; *P*=.9911) predominated over influenza B (23.8% and 29.6%, respectively; *P*=.5851) in both the hospital and Sentinel populations until March 2015 (Fig. 1C). Influenza B strains then became more abundant until the end of Sentinel surveillance in week 16 (data not shown).

**Table 1:** Demographics of hospital (without nosocomial cases) and Sentinel populations screened for influenza

Samples and individuals	Hospital samples	Sentinel samples
Total of samples*individuals*	2457*2457*	937*933* a
Positive samples*individuals*	442 (18%)*442*	487 (52%)*487*
Negative samples*individuals*	2015 (82%)*2015*	450^a^ (48%)*446*
Ratio positive/negative samples	0.22	1.08
Sex		
Male	1221	468^a^
Female	1236	465^a^
Ratio male/female	0.99	1.01
Age groups distribution (y)^b^		
0–4	168 *(6.8%)*	101 *(10.8%)*
5–14	63 *(2.6%)*	140 *(15%)*
15–29	106 *(4.3%)*	179 *(19.2%)*
30–64^a^	702 *(28.6%)*	439 *(47.1%)*
≥65	1418 *(57.7%)*	72 *(7.7%)*

a One female and three males had two distinct samples.

b Age information was lacking for two individuals among the Sentinel group.

Italic values are displayed to improve table legibility.

**Figure 2:**
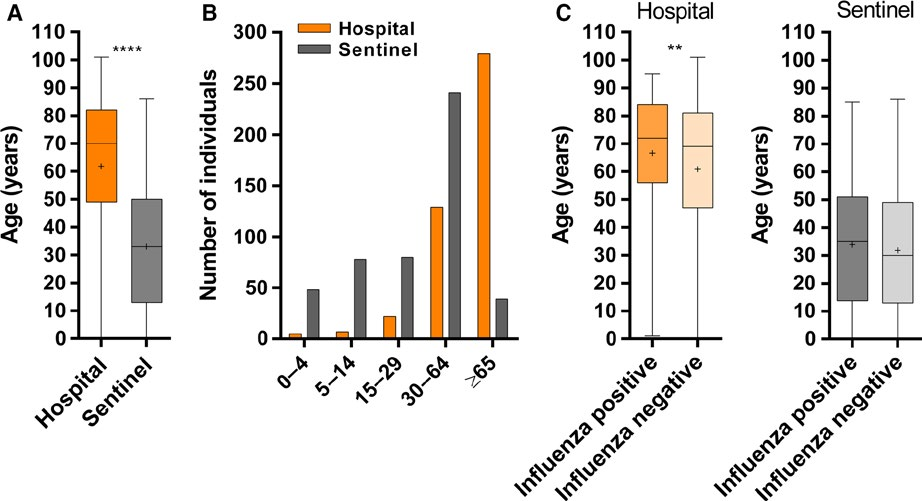
Age distribution of study individuals. (A) Age distribution of Sentinel and hospital individuals. (B) Number of Sentinel and hospital individuals per age group. (C) Age distribution of Sentinel and hospital individuals with positive and negative samples for influenza. Nosocomial influenza cases were not included in graphics (A) to (C). ****P<.0001; **P=.001; +: mean

The hospital population was older (median 70; range 0–101 years) than the Sentinel population (median 33; range 0–86 years); *P*<.0001 (Fig. 2A). When compared to sentinel population, young individuals were underrepresented in the hospital population (Fig. 2B). Although the total number of individuals tested in the two populations was different (Table 1; Fig. 1), similar proportions of males and females were observed (Table 1). Among the hospital and Sentinel populations, females (median 73 years; range 0–101 years, and median 36 years; range 0–86 years, respectively) were older than males (median 66 years; range 0–101 years, and median 29 years; range 0–83 years, respectively); *P*<.0001 (data not shown). Hospital patients with positive samples for influenza (median 72 years; range 1–95 years) were older than those with negative samples (median 69 years; range 0–101 years), *P*=.001 (Fig. 2C), particularly females. Even if not significant, a similar trend could be observed for Sentinel individuals.

**Figure 3:**
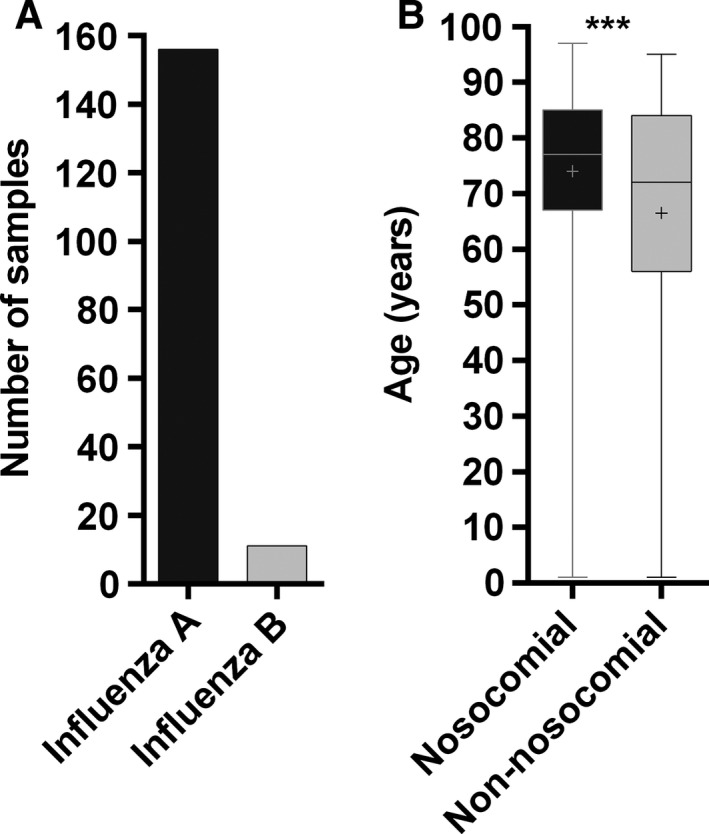
Nosocomial vs community‐acquired cases. (A) Number of nosocomial influenza A‐and B‐positive samples. (B) Age distribution of nosocomial and non‐nosocomial cases. +: mean; ***P=.0004

**Figure 4:**
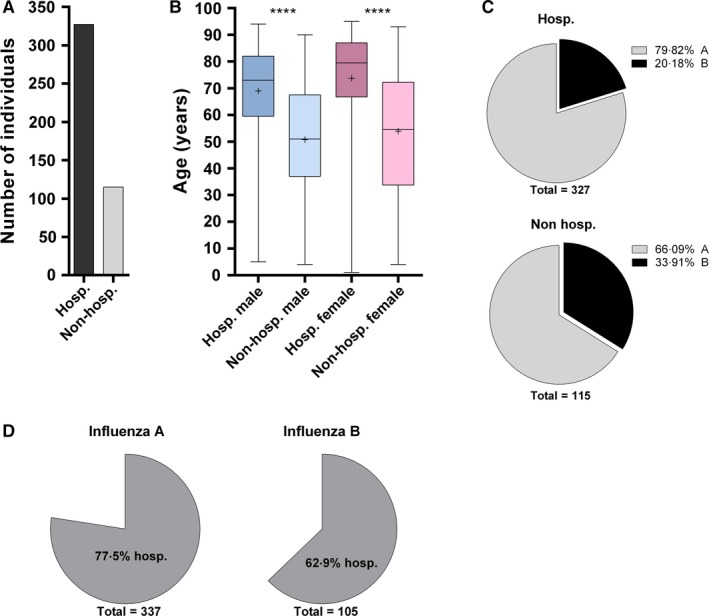
Hospitalized vs non‐hospitalized individuals with positive influenza samples in hospital population. (A) Number of individuals with a positive influenza sample who did or did not require hospitalization. (B) Age distribution of males and females among individuals who did or did not require hospitalization. (C) Percentage of influenza A and B among hospitalized (hosp) and non‐hospitalized (non‐hosp) patients. (D) Percentage of hospitalized (hosp) and non‐hospitalized (non‐hosp) patients among the total number of influenza A and B cases. Nosocomial influenza cases were not included in graphics (A) to (C). ****P<.0001; +: mean

**Figure 5:**
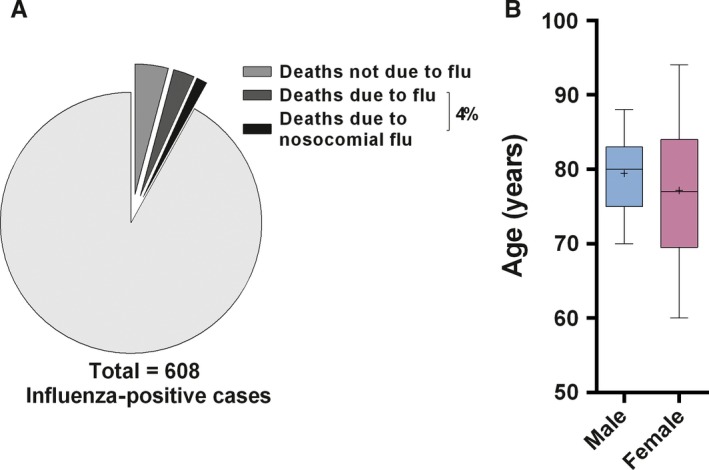
Deaths in the presence of influenza infections in the hospital population. (A) Number of deaths not due to an influenza infection (4.1%), deaths due to influenza including nosocomial cases (4% in total) among influenza‐positive cases. (B) Male and female age distribution for deaths probably due to an influenza infection (n=24). +: mean; noso: nosocomial

When focusing on the hospital population, among the 167 nosocomial influenza cases identified (68 males; 98 females), 93.4% carried an influenza A and 6.6% an influenza B strain (Table S3; Fig. 3A), representing 31.6% and 9.5% of the total A and B strains isolated in the hospital during the Influenza season, respectively. Interestingly, influenza A/B ratio was significantly different between nosocomial and non‐nosocomial cases (*P*<.0001, 95% CI 0.1018–0.2416). The nosocomial cases corresponded to 27.4% of the total influenza‐positive individuals, with the highest prevalence (30.4%) observed during January and February 2015 (weeks 1–8, data not shown). Individuals with nosocomial influenza (median 77; range 1–97 years) were older than non‐nosocomial cases (median 72; range 1–95 years; *P*=.0004, respectively) (Fig. 3B). One hundred and fifteen individuals (115/442 [26%]; 53 males and 62 females) had a positive influenza test, but were not hospitalized at the time of respiratory screening (e.g., outpatients consulting in emergency and ambulatory units), (Fig. 4A). Non‐hospitalized male and female individuals had a similar age distribution (median 51 years; range 4–90 years vs median 54.5 years; range 4–93 years, respectively; *P*>.999, data not shown), but they were significantly younger than hospitalized patients (median 73 years; range 5–94 years vs median 79.5 years; range 1–95 years; *P*<.0001, respectively) (Fig. 4B). Among patients who required hospitalization or who were already hospitalized but non‐nosocomial, 261 had influenza A and 66 had influenza B. This corresponded to 77.5% and 62.9% of the total respective strains and to 79.8% and 20.2%, respectively, of the influenza A and B viruses found in patients hospitalized on the day of sampling (Fig. 4C; Table S3). Interestingly, influenza A/B ratio was significantly different between patients who were hospitalized or required hospitalization and those who did not (*P*=.0044, 95% CI 0.04978–0.2420). A total of 49 deaths were reported among the 608 positive influenza cases identified at hospital (Fig. 5A). Twenty‐five deaths were considered non‐attributable to influenza infection, either because death occurred >30 days after respiratory sampling or on the basis of the available clinical information. Among the remaining 24 deaths, corresponding to 4% of all influenza‐positive individuals, 13 occurred in females (median 77 years; range 60–94 years) and 11 in males (median 80 years; range 70–88 years) of similar age, *P*=.5393 (Fig. 5A,B). Eight deaths occurred in patients with nosocomial influenza infections. Twenty influenza A and four influenza B viruses were isolated in fatal cases (data not shown). There was no significant association between influenza type and death (odds ratio 1.184; 95% CI 0.3967–3.533). No information on cases of death associated with influenza was available specifically for the Sentinel population.

### 3.2 Influenza subtypes and phylogenetic analysis of Sentinel and hospital samples

**Figure 6:**
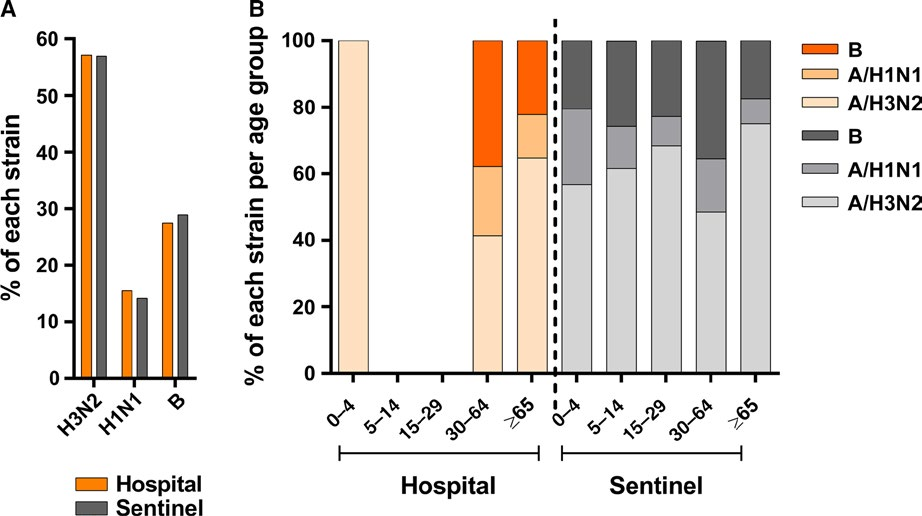
Influenza type and subtype distribution per population and per age group. (A) Percentage of H3N2, H1N1pdm09 (H1N1) and B strains in hospital and Sentinel populations; (B) among age groups. Nosocomial influenza cases were not included in graphics (A) and (B). Hospital, n=84; Sentinel n=478

One sample out of five was randomly chosen among the positive samples (n=609) isolated at the hospital (n=121) for phylogenetic characterization. Influenza A samples were further subtyped. Influenza strains from non‐nosocomial samples were then compared to those isolated from Sentinel samples. In total, 116 of the 121 samples could be subtyped, and thereof, 84 were non‐nosocomial. Forty‐eight were H3N2 (57.1%), 13 H1N1pdm09 (15.5%), and 23 B strains (27.4%). Similar proportions were observed for Sentinel samples with 56.9% of H3N2, 14.2% of H1N1pdm09, and 28.9% B strains (n=478) (Fig. 6A). Influenza subtype distribution within the age groups of 30–64 years and ≥65 years was similar for the hospital and Sentinel populations. The 0‐ to 29‐year individuals were underrepresented in the hospital population when compared to sentinel (Figs 2B and 6B). Among the 84 non‐nosocomial positive samples retained for genetic characterization in the hospital population, 59 had cycle threshold values <30 and were further sequenced (33 H3N2, 11 H1N1pdm09, and 15 B Yamagata lineage). All the sequenced influenza strains clustered within phylogenetic clades already observed for the Sentinel cases (Figs S1–S3). The H1N1pdm09 strains belonged to the 6B cluster and were antigenically similar to the H1N1pdm09 vaccine strain A/California/07/2009‐like (Fig. 1). The H3N2 strains were distributed among 3C.2, mainly 3C.2a, and 3C.3 clades (Fig. S2). Strains from these two clades are considered as antigenically distinct from the A/Texas/50/2012‐like vaccine strain.^14^ Influenza B strains were all found within clade 3 of the B/Yamagata/16/1988‐lineage, to which the predominant viruses belonged (Fig. S3) and matched with the seasonal Yamagata B/Massachusetts/2/2012‐like vaccine strain. As for Sentinel samples, we observed a B Yamagata (HA)‐Victoria (NA) reassortant (Fig. S3, A/Switzerland/17848/2015) among hospital samples. Finally, 26 nosocomial isolates had cycle threshold values <30 and were sequenced. The corresponding strains perfectly clustered with those found in the community (Figs S1–S3).

## 4 Discussion

Our results show a higher ratio of positive to negative samples for the Sentinel population compared to hospital patients. This observation could be explained by the fact that the “source” individuals for Sentinel samples were screened on an acute respiratory illness/ILI syndromic basis to specifically optimize the detection of influenza infections. This contrasts with hospital‐admitted patients who may present a larger spectrum of diseases and whose samples were collected for the screening of respiratory viruses in general, including influenza. Therefore, the probability to obtain a positive sample from a Sentinel individual was expected to be higher. Of note, acute respiratory illness/ILI‐based screening may limit the identification of cases with atypical symptoms either due to host immune status and/or virus strain‐specific characteristics.^15^

A similar proportion of influenza A and B strains co‐circulated in the hospital and Sentinel populations, with a predominance of influenza A/H3N2 virus. However, during March, influenza B virus dominated influenza A detection in both populations. This observation was consistent with data available for other European countries,^16^ USA^17^ and Canada.^18^

The general timing of influenza detection during the 2014–2015 season was similar for both populations. Interestingly, the number of influenza cases detected in hospital started to increase 2 weeks earlier than in Sentinel. This observation was in line with data from the South Korean surveillance systems, where the hospital‐based system detected the beginning of the influenza epidemic around one week before the national sentinel system^6^. The earlier influenza detection observed in the hospital population may be directly related to the higher number of samples screened in hospital but may also coincides with a decrease of activity of any surveillance network during Christmas holidays. In fact, individuals, who may normally have consulted a Sentinel practitioner, may have been redirected to hospital and non‐Sentinel primary care practices.

No significant genetic differences could be observed between influenza strains circulating in hospital and Sentinel populations. Influenza strain comparison was based on the HA1 gene, one of the main drivers in influenza pathogenicity. Nonetheless, influenza viral particles contain eight segments and the particular constellation of these genes is the major virulence determinant of each strain.^19^ Thus, we cannot exclude that significant differences may be found on other influenza genes than HA.

In contrast to the age groups of 0–4, 5–14, 15–29, and the 30–64 years that matched with the Swiss age structure, a low representation of the ≥65‐year age group was observed in the Sentinel population. The present study does not pinpoint a specific explanation for the latter observation. We observed that hospital patients for whom a respiratory panel was performed were older than Sentinel individuals. However, rapid diagnostic test results that are mainly used in the hospital for influenza identification in pediatric units (0–15 years old) were not included in our study, thus creating a detection bias toward adults and the elderly. In addition, in healthy adults without underlying health conditions, the molecular screening (PCR) for influenza is not systematically performed. It is well known that elderly individuals (≥65 years old) are also more prone to suffer from age‐related comorbidities and thus to develop a more severe influenza infection requiring hospital admission than younger adults.^20^, ^21^ Similarly, individuals with a positive influenza sample who required hospitalization at the day of sampling were also older than those who were not hospitalized. No significant association was found between the influenza type carried at the time of hospital admission and subsequent hospitalization. Of note, even if not significant, a trend toward a higher proportion of influenza B strains could be observed in the Sentinel 30‐ to 64‐year‐old age group. However, we keep in mind that few samples were typed per age group in the hospital population.

Sentinel and hospital females were older than males, which may be explained by their higher lifespan expectancy in Switzerland.^22^ Twenty‐four deaths associated with influenza or subsequent secondary infections were reported during the season at our hospital, but these were equally distributed among both sexes. Although influenza A was found in most fatal cases, no significant association could be identified between the influenza type and death.

Approximately 1 of 4 influenza infections identified in hospital during the 2014–2015 season was nosocomial, with a higher proportion during the epidemic period. Several factors could account for this observation, such as low vaccine uptake and suboptimal vaccine efficiency due to antigenic drift as was the case for this season, particularly for H3N2 strains, but also insufficient compliance with infection prevention and containment measures by healthcare workers, patients, and visitors.^23^, ^24^

Our study has some limitations. As the study was retrospective, some demographic and epidemiological data that would be interesting to compare between both populations were either unavailable or incomplete. Notably, influenza‐associated comorbidity information was not reliably reported for the Sentinel population and mortality data were missing. Among the hospital population, children and young adults were underrepresented, while the elderly may possibly be underrepresented in the Sentinel data. Of note, this study was conducted only during the 2014–2015 flu season in a single center. Finally, the comparability between the single‐locality hospital‐based data and country‐wide Sentinel data relies on similar influenza epidemiological patterns in the area served by the hospital and in the country as a whole. Although this could not be demonstrated with the available Sentinel data, a similar time‐course and similar proportions of influenza A and B viruses are suggestive that the comparison is meaningful.

Our results confirm that the Swiss Sentinel for influenza surveillance is adequate to detect the onset of influenza epidemics and to obtain an accurate overview of the circulating strains necessary for the correct identification of future vaccine strains. Nevertheless, it is not optimal to estimate the severity of the circulating strains and to assess the impact of influenza on high‐risk populations. This issue could be addressed by the monitoring of severe acute respiratory illness in hospitals with laboratory confirmation for influenza virus, as proposed by the WHO^25^, but not implemented in Switzerland so far.

We conclude that a hospital‐based system for influenza surveillance could be a useful complement to the current Sentinel system. This upgraded system would have several advantages as a greater number of individuals for syndromic screening in a single medical setting (i.e., one major hospital per Swiss sentinel region), the possibility to draw a more accurate clinical picture of influenza across different age groups, particularly in the case of the emergence of a new or more severe influenza strains, and a better follow‐up of influenza‐associated comorbidities and deaths. Severe acute respiratory illness surveillance would be implementable in hospital Sentinel settings with limited additional costs. Accurate detection of circulating influenza strains would be conserved. The latter information would be crucial for the accurate selection of the influenza vaccine strains, which is a major goal of influenza surveillance worldwide. Additional prospective studies conducted in different medical settings across Switzerland are required during future influenza seasons to validate and complement our observations.
