# Supplementary figures and images for: Hospital surveillance of influenza strains: a concordant image of viruses identified by the Swiss Sentinel system?
Source: Influenza Other Respir Viruses. 2016 Aug 31;11(1):41–7. doi: 10.1111/irv.12417 (PMC5155643; doi:10.1111/irv.12417)

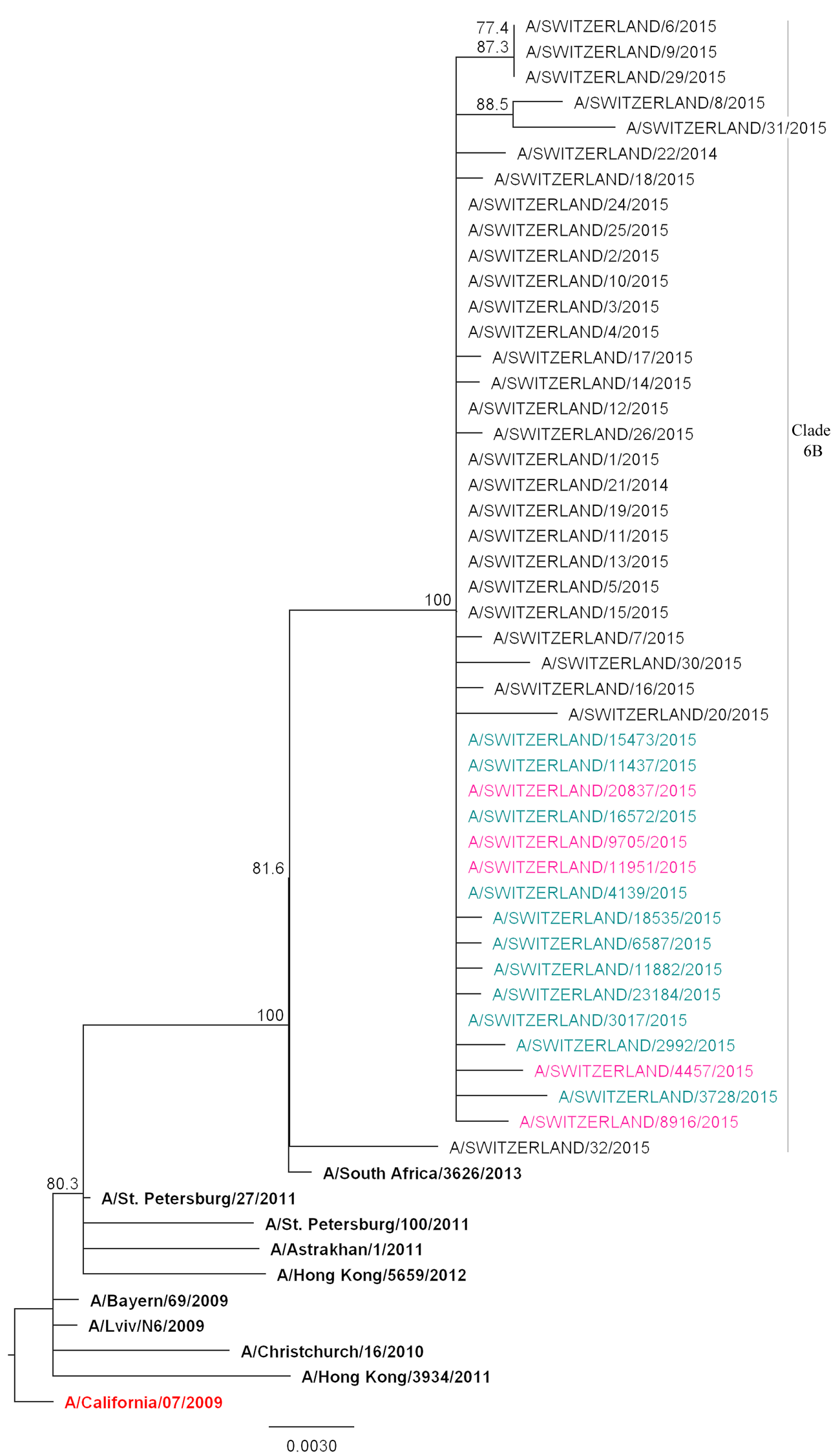

Supplement: Supplementary file 1 [file IRV-11-41-s001.tiff]

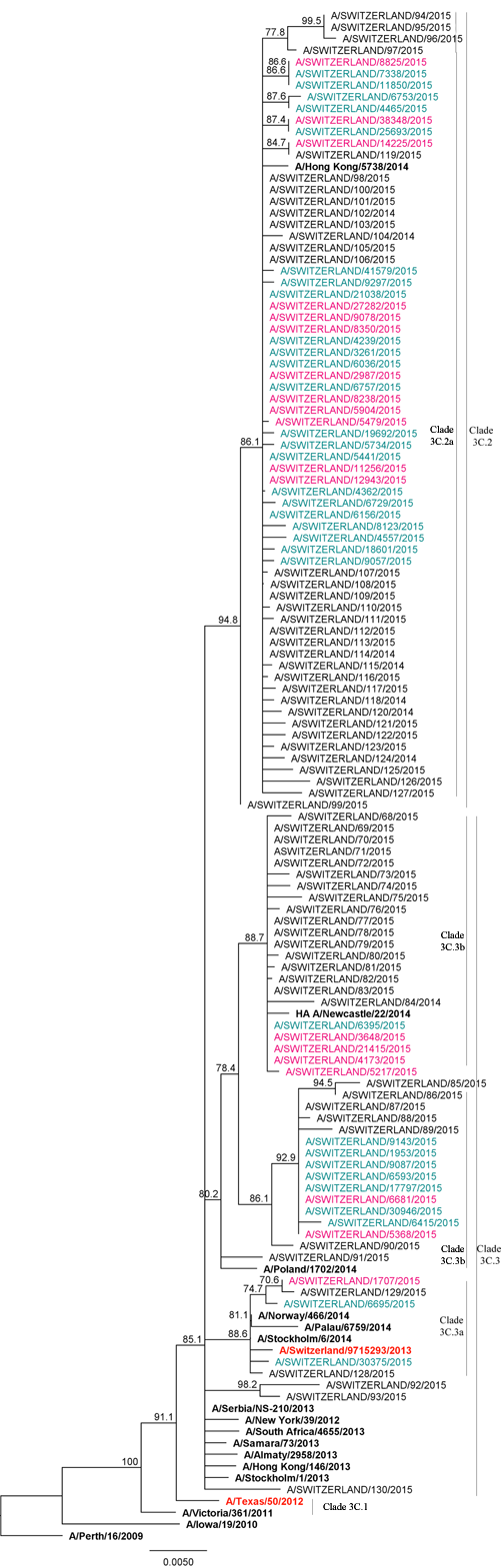

Supplement: Supplementary file 2 [file IRV-11-41-s002.tiff]

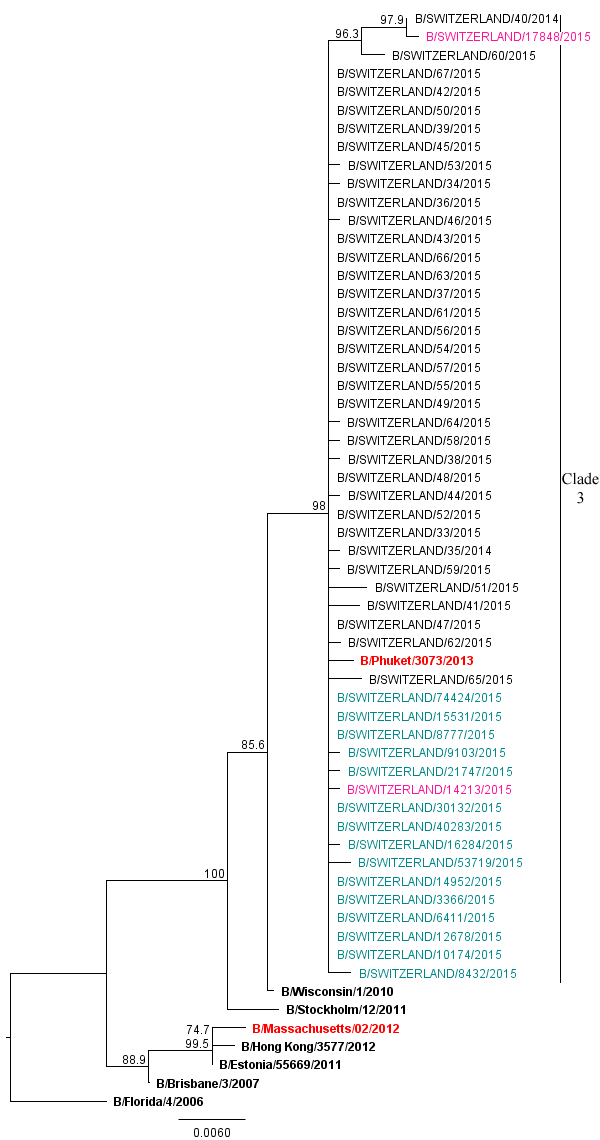

Supplement: Supplementary file 3 [file IRV-11-41-s003.tiff]
