# Supplementary material for: Hospital surveillance of influenza strains: a concordant image of viruses identified by the Swiss Sentinel system?
Source: Influenza Other Respir Viruses. 2016 Aug 31;11(1):41–7. doi: 10.1111/irv.12417 (PMC5155643; doi:10.1111/irv.12417)
Supplement: Supplementary file 4 [file IRV-11-41-s004.docx]

**Supporting information**

**Figures legends**

Supplementary Figure 1. Phylogenetic analysis of the HA1 gene of A/H1N1pdm09-like viruses. In red, 2014-2015 vaccine strain. Hospital strains are in pink (nosocomial cases) and green (non-nosocomial cases); Sentinel strains are in black. Bold black corresponds to reference strains provided by the United Kingdom Medical Research Council-National Institute for Medical Research. Sentinel n=29 and Hospital n=16.

Supplementary Figure 2 . Phylogenetic analysis of the HA1 gene of A/H3N2 viruses. In red, 2014-2015 vaccine strain. Hospital strains are in pink (nosocomial cases) and green (non-nosocomial cases); Sentinel strains are in black. Bold black corresponds to reference strains as provided by the United Kingdom Medical Research Council-National Institute for Medical Research. Sentinel n=62 and Hospital n=51.

Supplementary Figure 3. Phylogenetic analysis of the HA1 gene of B Yamagata lineage viruses. In red, 2014-2015 vaccine strain. Hospital strains are in pink (nosocomial cases) and green (non-nosocomial cases); Sentinel strains are in black. Bold black corresponds to reference strains as provided by the United Kingdom Medical Research Council-National Institute for Medical Research. Sentinel n=35 and Hospital n=17.

**Tables**

**Supplementary Table 1.** Influenza virus rtRT-PCR and sequencing specific primers/probes

| **Primer type** | **Target** | **Primer name** | **Primer/probe sequences (5’-3’)** |
| --- | --- | --- | --- |
| RT | Influenza A | Uni12W | AGCRAAAGCAGG |
|  | Influenza B | BUni11w | AGCAGAAGCGS |
| Screening one-step rtRT-PCR | Influenza A MP | InfA-CDC For | GACCRATCCTGTCAC CTCTGAC |
|  |  | InfA-CDC Rev | AGGGCATTYTGGACAAAKCGTCTA |
|  |  | InfA Pro CDC | FAM-TGCAGTCCTCGCTCACTGGGCACG-BHQ1 |
| Screening one-step rtRT-PCR | Influenza B NS | BcdcFor | TCCTCAAYTCACTCTTCGAGCG |
|  |  | BcdcRev | CGGTGCTCTTGACCAAATTGG |
|  |  | BcdcPro | Yaki-CCAATTCGAGCAGCTGAAACTGCGGTG-BHQ |
| Influenza A subtyping | Influenza A HA3 | AH3cdcFor | AAGCATTCCYAATGACAAACC |
|  |  | AH3cdcRev | ATTGCRCCRAATATGCCTCTAGT |
|  |  | AH3cdcPro | FAM-CAGGATCACATATGGGSCCTGTCCCAG-BHQ |
| One-step rtRT-PCR | Influenza A HA1 | SwiH1 F | GGGTAGCCCCATTGCATTT |
|  |  | SwiH1 R | TGGAGAGTGATTCACACTCTGGAT |
|  |  | SwiH1Ge P | FAM-AAYATTGCTGGCTGGATCCTGGGA-BHQ |
| Sequencing | Influenza A HA3 | **AH3G** | AAGCAGGGGATAATTCTATT |
|  |  | **AH3H** | ATGCCTGAAACCGTACCAAC |
|  |  | AH3CII* | GCTTCCATTTGGAGTGATGC |
|  |  | AH3B | AGCAAAGCTTACAGCAACTG |
|  |  | AH3I* | TCCCTCCCAACCATTTTCT |
|  |  | AH3F567* | TTGAACGTGACTATGCCAAACAAT |
|  |  | AH3R650* | TTGGTCACTGTCCGTACTCGGGTG |
|  | Influenza A HA1 | **AH1pdmF1** | AGCAAAAGCAGGGGAAAACAAAAGC |
|  |  | **AH1p1313R** | CCAGGAAACCATCATCAACT |
|  |  | cswHAF31 | ATGAAGGCAATACTAGTAGTTCTGC |
|  |  | AH1p873R* | CAGATCCAGCATTTCTTTCCATTGT |
|  |  | cswHAF451 | GCCCAATCATGACTCGAACAAAGG ь |
|  |  | AH1p1263R* | CCACTGCTGTGAACTGTGTATTC |
|  |  | cswHA475R* | CCTTTGTTCGAGTCATGATTGGGC |
|  |  | AH1p848F* | ACAATGGAAAGAAATGCTGGATCTG |
|  | Influenza B HA | **BHA1F1** | AATATCCACAAAATGAAGGCAATA |
|  |  | **BHA1R1** | ATCATTCCTTCCCATCCTCCTTCC |
|  |  | BHAF | CCAGCAATAGCTCCGAAGAA |
|  |  | BHA25 | CCACAAAATGAAGGCAATAA |
|  |  | BHAF458* | AGAAAAGGCACCAGGAGGACCCTA |
|  |  | BHAR652* | GGAACCCCCAAACAGTAATTTGGT |

In bold: first PCR primers, in green and/or violet: nested (B) or hemi-nested (H1 and H3) amplification PCRs.* primers used for Sanger sequencing.

Supplementary Table 2. Sequences chosen as references for the respective phylogenic trees.

**Supplementary Table 3.** Virological data for hospital-based positive samples

| **Influenza types** | | **A** | **B** | **Total** |
| --- | --- | --- | --- | --- |
| *Total number* | | 493 | 116 | 609* |
| **Samples “origin”** | | | | **Total** |
| *Community* | *Hospitalization not required* | 76 | 39 | 115 *(18.9%)* |
|  | *Hospitalization required* | 261 | 66 | 327 *(53.7%)* |
| *Nosocomial* | | 156 | 11 | 167 (27.4%) |

**For 608 individuals.*
